# Recurrent Adipsic Hypernatremia in a Fully Independent Non-psychiatric Patient With Multiple Congenital Anomalies: A Case Report

**DOI:** 10.7759/cureus.23942

**Published:** 2022-04-08

**Authors:** Rakan B Alanazi, Moustafa S Alhamadh, Sultan T Alqarni, Khaled H Alanazi, Basel Alheijani

**Affiliations:** 1 Medicine, College of Medicine, King Saud Bin Abdulaziz University for Health Sciences, Riyadh, SAU; 2 Surgery, College of Medicine, King Saud Bin Abdulaziz University for Health Sciences, Riyadh, SAU; 3 Internal Medicine, King Abdulaziz Medical City, Riyadh, SAU; 4 Internal Medicine, College of Medicine, King Saud Bin Abdulaziz University for Health Sciences, Riyadh, SAU

**Keywords:** altered mental status, psychogenic adipsia, osmolality, adipsia, hypernatremia

## Abstract

Osmoregulation is a fundamental process of homeostasis that maintains metabolic and biochemical reactions, thermoregulation, and fluid-electrolytes balance. Fluid-electrolytes imbalance leads to various clinical manifestations ranging from mild weakness to severe neurological dysfunction. Adipsic hypernatremia is an exceedingly rare life-threatening condition characterized by defective osmoregulatory mechanisms. It is more often reported in patients with severe untreated psychiatric disorders for unknown etiologies, but it may result from congenital or acquired hypothalamic lesions in the form of stroke, neoplastic infiltration, trauma, or infection. Herein we report an unusual case of isolated hypernatremia in a fully independent non-psychiatric 27-year-old diabetic male with spina bifida, repaired cleft palate, and mild caudal regression syndrome.

## Introduction

Osmoregulation is a fundamental process of homeostasis that ensures sufficient organs perfusion, thermoregulation, excretion of toxins, and electrolyte balance. This sophisticated process is regulated by different organs systems with the kidneys being preeminent via controlling fluid and electrolytes retention and excretion, but other mechanisms like hormonal interactions are also implicated [1,2]. At a higher level, the hypothalamus stimulates antidiuretic hormone (ADH) release in response to increased body fluid osmolarity to modulate fluid reabsorption in the nephrons, decreasing fluid osmolarity [2]. Perturbation of fluid and electrolytes balance leads to various manifestations such as confusion with altered mental status, seizure, weakness, headache, and oliguria-anuria [3,4]. Hypernatremia is one of the most frequently encountered electrolyte abnormalities with an estimated mortality that can exceed 60% [5]. It develops as a result of insufficient fluid intake or increased fluid loss as seen in severe cases of vomiting and diarrhea. Consequently, hypothalamic osmoreceptors sense and, thus, stimulate thirst via several autonomic and hormonal mechanisms [6]. Adipsic hypernatremia is an extremely rare life-threatening cause of hypernatremia characterized by defective osmoregulatory mechanisms of thirst and ADH secretion [7]. It occurs more often in patients with severe psychiatric disorders, but it may result from congenital or acquired hypothalamic lesions, in the form of stoke, neoplastic infiltration, trauma or infection, or autoantibodies against the circumventricular organs [8,9]. In this article, we report a case of adipsic hypernatremia in a 27-year-old diabetic male without history of psychiatric disorder or head trauma.

## Case presentation

A 27-year-old male, a known case of type 1 diabetes mellitus, spina bifida, repaired cleft palate, and caudal regression syndrome, was brought to our emergency department by his family due to a three-day history of fatigue and decreased oral intake. Upon arrival, he was conscious but confused, ill-appearing, severely dehydrated, and unkempt with ragged clothes on. History could not be taken from him as he was only moaning and could not communicate well.

According to the family, the patient was fully independent, and he has been working in his father’s firm. He was in his usual state of health until three to five days ago when he stopped eating and drinking and later on complained of generalized fatigue with weakness in his lower limbs, restricting his ability to walk without obvious preceding event. The patient’s brother stated that the patient was not compliant with his insulin as he forgets to take them. He also described having to remind him to drink water sometimes as his lips appeared cracked and dry. The family denied having a similar condition. His past medical history was notable for poor glycemic control and multiple admissions for decreased oral intake and severe hypernatremia without evidence of psychiatric disorder or other causes, such as gastric lose, diabetes insipidus (DI), or diabetic ketoacidosis (DKA), last one of which was three weeks ago.

On examination, the patient was clearly confused and had multiple lacerations throughout his body with perianal skin excoriation and mold-like lesion, suspicious of fungal infection. He was hypotensive (109/67 mmHg) and tachycardiac (106 beats per minute) with a Glasgow Coma Scale of 14. The patient’s mouth was dry, and his extremities were cold with delayed capillary refill. His radial pulses were faint with a high rate but normal rhythm. His pupils were equally round and reactive to light, and his cardiovascular examination was unremarkable with normal S1 and S2 and no added sounds or murmurs. The patient’s chest was clear with equal bilateral air entry and normal vesicular breathing. His abdomen was diffusely tender to palpation without appreciable masses or evidence of organomegaly. His anal sphincter tone was normal with no fecal incontinence. Neurological examination was only remarkable for bilateral lower limb weakness (3/5) with negative Babinski sign with normal sensation, perception, and reflexes in upper and lower limbs.

Laboratory investigations were consistent with hyperglycemic (random blood glucose {RBG}: 28.6 mmol/L {2.9-7.8 mmol/L}), thrombocytopenia (57 × 10^9^/L {150-400 × 10^9^/L}), kidney injury (serum creatinine {Cr}: 141 umol/L {64-110 umol/L}, blood urea nitrogen {BUN}: 24.4 mmol/L {3.2-7.4 mmol/L}), transaminitis (aspartate aminotransferase {AST}: 105 U/L {5-34 U/L}, alanine transaminase {ALT}: 93 U/L {5-55 U/L}, alkaline phosphatase {ALP}: 235 U/L {30-120 IU/L}), hyperosmolar hypernatremia (Na: 196 mmol/L {136-145 mmol/L}, serum osmolarity: 450 mOsm/kg {275 to 295 mOsm/kg}), hyperchloremia (150 mmol/L {98-107 mmol/L}), hypercalcemia (2.65 mmol/L {2.1-2.55 mmol/L}), hypermagnesemia (1.45 mmol/L {0.66-1.07 mmol/L}), hypokalemia (3.1 mmol/L {3.5-5.1 mmol/L}), hypophosphatemia (0.45 mmol/L {0.74-1.52 mmol/L}), and elevated lactate (4.89 mmol/L {0.5-2.2 mmol/L}) and uric acids (805 umol/L {220-450 umol/L}) (Table 1). Arterial blood gas analysis was done and revealed normal pH (7.38 {7.35-7.45}) with normal pCO2 (42.2 mmHg {35-45 mmHg}) and bicarbonate (24.8 mEq/L {22-26 mEq/L}) levels. Hemoglobin A1c (HbA1c) was not done at the most recent visit, but his previous readings have been always high, ranging from 7.3-11.2% (4-5.6%). Urinalysis was notable for high specific gravity (1.053), glucose, mild ketones, and blood. Further evaluation revealed urine osmolarity of 958 mOsm/kg.

**Table 1:** Important laboratory values during the period of admission in the interval of two days. BUN: blood urea nitrogen

Variables	Reference value	On admission	Day 3	Day 5	On discharge
Calcium (mmol/L)	2.1-2.55 mmol/L	2.65	1.77	1.84	1.97
Sodium (mmol/L)	136-145 mmol/L	196	148	144	149
Chloride (mmol/L)	98-107 mmol/L	>150	116	112	114
Magnesium (mmol/L)	0.66-1.07 mmol/L	1.45	0.78	0.71	0.73
Potassium (mmol/L)	3.5-5.1 mmol/L	3.1	4.9	3.8	3.9
Phosphorus (mmol/L)	0.74-1.52 mmol/L	0.45	0.69	0.73	0.85
BUN (mmol/L)	3.2-7.4 mmol/L	24.4	14.9	4.4	2.2
Creatinine (umol/L)	64-110 umol/L	141	70	44	46
Uric acid (umol/L)	220-450 umol/L	805	568	212	253
Random blood glucose (mmol/L)	2.9-7.8 mmol/L	28.6	20.2	13.1	7.1

Chest radiograph was unremarkable, and brain computed tomography (CT) scan showed calcifications in the basal ganglia and posterior thalami, not suggestive of any known disorder or syndrome that might explain his condition. Additionally, the CT showed no signs of hypothalamic lesion, ischemic or hemorrhagic changes. MRI of the spine (cervical and thoracolumbar) was negative for cord compression. In a previous admission, MRI was done and showed focal signal abnormality in the splenium of the corpus callosum with no evidence of pituitary or circumventricular organs lesion.

On admission, he was hypotensive and dehydrated, thus, he was given 0.9% normal saline IV (1000 mL/h bolus) and admitted under internal medicine. He was immediately rehydrated with Ringer's lactate solution. Potassium was corrected with IV potassium phosphate and insulin was given to control the patient’s hyperglycemia. Nasogastric and feeding tubes were inserted, and to closely monitor the patient’s sodium level, a decision was made to admit him to the intensive care unit. Hypernatremia was corrected with water through nasogastric tube (NGT) and dextrose 5% in half (0.45%) normal saline at a rate of 8-12 mEq/L of sodium daily. After four days of admission, intravenous fluid was stopped, and free water intake was encouraged (150 mL every four hours). During admission and despite his cracked lips, he was not looking thirsty. This history was augmented by his family, who stated on multiple occasions that he wasn't asking for water for days.

After eight days of admission, he was vitally stable with no active complaint. His lower limbs weakness has completely resolved (5/5) and his Glasgow Coma Scale was 15/15. Discharge laboratory values showed significant improvement in his electrolytes (sodium: 149 mmol/L, potassium: 3.9 mmol/L, chloride: 114 mmol/L, calcium: 1.97 mmol/L, phosphate: 0.85 mmol/L, and magnesium: 0.73 mmol/L), kidney function (Cr: 46 umol/L, BUN: 2.2 mmol/L, uric acid: 253 umol/L), and glucose level (RBG: 7.1 mmol/L), and HbA1C level was not measured during this admission. He was discharged on insulin (glargine and aspart) and scheduled a follow-up with endocrinology and neurology.

## Discussion

Osmoregulation is an essential process to maintain metabolic and biochemical reactions, thermoregulation, and fluid-electrolytes balance. This sophisticated process is regulated by different organs systems with the kidneys being preeminent via controlling fluid and electrolytes retention and excretion from the glomerular filtrate [1]. When serum osmolarity increases, hypothalamic osmoreceptors, particularly supraoptic nuclei, synthesize ADH and transports it to the posterior pituitary gland through a carrier protein called neurophysin [2]. Once in the bloodstream, ADH binds the principal cells of the collecting duct, increasing aquaporins-2 channel and, therefore, water reabsorption in the nephrons [8,9]. Juxtaglomerular apparatus is also implicated in osmoregulation via renin-angiotensin-aldosterone system in response to low renal perfusion [10]. Any lesions involving this sophisticated system would impair osmoregulatory mechanisms.

Hypernatremia is the most prevalent electrolytes disturbance, with a mortality rate reaching up to 60% depending on the sodium level [7]. In this article, we reported an unusual case of recurrent hyperosmolar hypernatremia in a 27-year-old non-psychiatric diabetic male with multiple congenital anomalies attributed eventually to adipsic hypernatremia. Adipsic hypernatremia is an exceedingly rare life-threatening condition characterized by defective osmoregulatory mechanisms [11]. It is more often reported in patients with severe untreated psychiatric disorders of unknown etiologies, but it may result from congenital or acquired hypothalamic lesions, in the form of stroke, neoplastic infiltration, trauma or infection, or autoantibodies against the circumventricular organs, particularly the subfornical organ [12-15].

It is worth mentioning that this patient’s recent presentation is suspicious for abuse or neglect. As mentioned above, in the case presentation, the patient appearance was unkempt, and his clothes were ragged. Upon examination, multiple diffuse bruises and perianal mold-like lesion were seen, all of which raise concern for abuse or neglect. Social workers have been involved in this patient’s care, but abuse or neglect could not be confirmed or excluded. Psychiatry was consulted to see the patient, but the patient did not meet any Diagnostic and Statistical Manual of Mental Disorders, Fifth Edition (DSM-5) diagnostic criteria.

To the best of our knowledge, only a few cases of adipsic hypernatremia without evidence of causative illness have been previously reported [8]. We believe that this case is interesting for the following reasons: (1) this is the first case of adipsic hypernatremia in the Kingdom of Saudi Arabia. (2) This case cannot be explained by acquired causes as no intracranial infection nor hypothalamic, or circumventricular organs lesion were found, and the patient, as well as his family, denied history of head trauma. Although his brain CT and MRI showed calcifications in the basal ganglia and posterior thalami, focal signal abnormality in the splenium of the corpus callosum, respectively, these were not suggestive nor showed any abnormalities that might explain his condition. Furthermore, these symptoms and the absence of radiological findings for his condition might be caused by cellular damage in the subfornical organ tissue by immune response, however, patients with subfornical organ damage tend to be obese and young [16]. For our patient, he has a normal BMI (22.2) and his symptoms started in his 20s, but he had no assay for autoantibodies targeting the sensory circumventricular organs, thus it could not be excluded nor confirmed. In addition, the patient’s urine osmolarity was extremely elevated without polyuria, which makes the diagnosis of DI unlikely. Moreover, although urine analysis showed mild ketone bodies, the patient’s ABG revealed normal pH ruling out DKA. Even though hyperosmolar hyperglycemic state (HHS) cannot be fully ruled out, the patient is young type 1 diabetic with mild ketonuria, which is very unusual for HHS. However, it is important to keep in mind that the patient’s presentation was highly suspicious for abuse or neglect. (3) Although this patient has multiple congenital anomalies, including spina bifida, cleft palate, and caudal regression syndrome with sacrococcygeal agenesis and mild truncation of the spinal cord, to the best of our knowledge, none has an association with adipsia or hypothalamus-pituitary-axis disturbances.

## Conclusions

Adipsic hypernatremia is a rare life-threatening condition characterized by impaired osmoregulation secondary to congenital or acquired hypothalamic lesion in the form of stroke, neoplastic infiltration, trauma, or cerebral infection. Ruling out serious acquired causes must be followed by a thorough psychiatric evaluation as this disease is often seen with untreated psychiatric disorders.

## References

[1] Larsen EH, Deaton LE, Onken H, O'Donnell M, Grosell M, Dantzler WH, Weihrauch D (2014). Osmoregulation and excretion. Compr Physiol.

[2] Chen J, Sabir S, Al Khalili Y (2021). Physiology, osmoregulation and excretion. StatPearls [Internet].

[3] Balcı AK, Koksal O, Kose A, Armagan E, Ozdemir F, Inal T, Oner N (2013). General characteristics of patients with electrolyte imbalance admitted to emergency department. World J Emerg Med.

[4] Wilson RF, Sibbald WJ (1976). Fluid and electrolyte problems in the emergency department. JACEP.

[5] Phillips MG, Gabow PA (1990). Psychogenic adipsia in a patient with psychotic depression. Am J Kidney Dis.

[6] Chothia MY, George K, Sheik M, Davids MR (2018). Hypodipsic-hypernatremia syndrome in an adult with polycythemia: a case report. J Med Case Rep.

[7] Robertson GL (1991). Disorders of thirst in man. Thirst. ILSI Human Nutrition Reviews.

[8] Rodriguez A, Fogelfeld L, Robertson G (2019). Hypernatremic hydrophobic transient adipsia without organic or severe psychiatric disorder. J Clin Endocrinol Metab.

[9] Desai PM, Mbachi C, Mathew M, Attar B, Mba B (2020). Psychogenic adipsia presenting as recurrent functional vomiting and hypernatremia. J Am Osteopath Assoc.

[10] Berliner RW, Levinsky NG, Davidson DG, Eden M (1958). Dilution and concentration of the urine and the action of antidiuretic hormone. Am J Med.

[11] Kurtzman NA, Boonjarern S (1975). Physiology of antidiuretic hormone and the interrelationship between the hormone and the kidney. Nephron.

[12] Skøtt O, Briggs JP, Lorenz JN, Weihprecht H (1990). On the intrarenal regulation of renin release from the juxtaglomerular apparatus. Kidney Int Suppl.

[13] Farley PC, Lau KY, Suba S (1986). Severe hypernatremia in a patient with psychiatric illness. Arch Intern Med.

[14] Harrington C, Grossman J, Richman K (2014). Psychogenic adipsia presenting as acute kidney injury: case report and review of disorders of sodium and water metabolism in psychiatric illness. Psychosomatics.

[15] Manning S, Shaffie R, Arora S (2017). Case report: severe hypernatremia from psychogenic adipsia. F1000Res.

[16] Utsunomiya AN, Hiyama TY, Okada S, Noda M, Kobayashi M (2017). Characteristic clinical features of adipsic hypernatremia patients with subfornical organ-targeting antibody. Clin Pediatr Endocrinol.

